# Mechanisms Underlying the Delayed Activation of the Cap1 Transcription Factor in *Candida albicans* following Combinatorial Oxidative and Cationic Stress Important for Phagocytic Potency

**DOI:** 10.1128/mBio.00331-16

**Published:** 2016-03-29

**Authors:** Iaroslava Kos, Miranda J. Patterson, Sadri Znaidi, Despoina Kaloriti, Alessandra da Silva Dantas, Carmen M. Herrero-de-Dios, Christophe d’Enfert, Alistair J. P. Brown, Janet Quinn

**Affiliations:** a Institute for Cell and Molecular Biosciences, Faculty of Medicine, Newcastle University, Newcastle upon Tyne, United Kingdom; b Institut Pasteur, Unité Biologie et Pathogénicité Fongiques, Département Mycologie, Paris, France; c INRA, USC2019, Paris, France; d School of Medical Sciences, University of Aberdeen, Aberdeen, United Kingdom

## Abstract

Following phagocytosis, microbes are exposed to an array of antimicrobial weapons that include reactive oxygen species (ROS) and cationic fluxes. This is significant as combinations of oxidative and cationic stresses are much more potent than the corresponding single stresses, triggering the synergistic killing of the fungal pathogen *Candida albicans* by “stress pathway interference.” Previously we demonstrated that combinatorial oxidative plus cationic stress triggers a dramatic increase in intracellular ROS levels compared to oxidative stress alone. Here we show that activation of Cap1, the major regulator of antioxidant gene expression in *C. albicans*, is significantly delayed in response to combinatorial stress treatments and to high levels of H_2_O_2_. Cap1 is normally oxidized in response to H_2_O_2_; this masks the nuclear export sequence, resulting in the rapid nuclear accumulation of Cap1 and the induction of Cap1-dependent genes. Here we demonstrate that following exposure of cells to combinatorial stress or to high levels of H_2_O_2_, Cap1 becomes trapped in a partially oxidized form, Cap1^OX-1^. Notably, Cap1-dependent gene expression is not induced when Cap1 is in this partially oxidized form. However, while Cap1^OX-1^ readily accumulates in the nucleus and binds to target genes following high-H_2_O_2_ stress, the nuclear accumulation of Cap1^OX-1^ following combinatorial H_2_O_2_ and NaCl stress is delayed due to a cationic stress-enhanced interaction with the Crm1 nuclear export factor. These findings define novel mechanisms that delay activation of the Cap1 transcription factor, thus preventing the rapid activation of the stress responses vital for the survival of *C. albicans* within the host.

## IMPORTANCE

Combinatorial stress-mediated synergistic killing represents a new unchartered area in the field of stress signaling. This phenomenon contrasts starkly with “stress cross-protection,” where exposure to one stress protects against subsequent exposure to a different stress. Previously we demonstrated that the pathogen *Candida albicans* is acutely sensitive to combinations of cationic and oxidative stresses, because the induction of H_2_O_2_-responsive genes is blocked in the presence of cationic stress. We reveal that this is due to novel mechanisms that delay activation of the Cap1 AP-1-like transcription factor, the major regulator of the H_2_O_2_-induced regulon. Cap1 becomes trapped in a partially oxidized form following simultaneous exposure to oxidative and cationic stresses. In addition, cationic stress promotes the interaction of Cap1 with the Crm1 nuclear export factor, thus inhibiting its nuclear accumulation. These mechanisms probably explain the potency of neutrophils, which employ multiple stresses to kill fungal pathogens.

## INTRODUCTION

*Candida albicans* is a major fungal pathogen of humans. Recent estimates indicate that invasive *C. albicans* infections are associated with disturbingly high mortality rates of between 46 and 75% and are responsible for over 400,000 life-threatening systemic infections each year (1). Immunocompromised patients are most at risk of systemic candidiasis, such as those receiving immunosuppressive treatments for cancer or transplant surgery. In contrast, in healthy hosts, robust immune protection mechanisms prevent such systemic infections, with innate immune cells such as macrophages and neutrophils providing the first line of defense.

A major antimicrobial defense mechanism mounted by innate immune cells is the production of superoxide anions (O_2_^−^) by the NADPH oxidase complex (2). The importance of this oxidative burst in fungal killing is exemplified by patients with chronic granulomatous disease (CGD). CGD is a genetic disorder in which patients have a defective phagocytic NADPH oxidase complex. These patients are significantly more susceptible to systemic *Candida* infections (3). The levels of O_2_^−^ generated by neutrophils in the phagocytic vacuole are estimated to range between 1 (4) and 4 mol liter^−1^ (5). The steady-state levels of O_2_^−^ are, however, likely to be much lower (4) due to its rapid dismutation to the more reactive hydrogen peroxide, H_2_O_2_ (6). Moreover, the resultant H_2_O_2_ can also generate hypochlorous acid (HOCl) by the action of myeloperoxidase. O_2_^−^ can also react with the nitric oxide radical, generated by nitric oxide synthase, to form peroxynitrite (ONOO^−^). Thus, the production of superoxide leads to the generation of a range of reactive oxygen, nitrogen, and chloride species (reviewed in references 4 and 6).

The prevailing view that reactive oxygen species (ROS) are a major factor underlying the fungicidal action of phagocytes does, however, conflict with previous studies which have demonstrated that *C. albicans* is more resistant to multiple oxidative stress-inducing agents than other fungi (7, 8). Following exposure to ROS, the activation of several pathways allows *C. albicans* to detoxify the stress and repair the oxidative damage to cellular components (9). One major mechanism involves the rapid induction of genes with antioxidant properties (10–14), and this is largely regulated by the AP-1-like transcription factor Cap1 (15, 16). Similar to the homologous *Saccharomyces cerevisiae* Yap1 and *Schizosaccharomyces pombe* Pap1 transcription factors (17, 18), H_2_O_2_-mediated-Cap1 activation is triggered by the oxidation of redox-active cysteine residues located within two cysteine-rich domains (n-CRD and c-CRD). Based on studies in *S. cerevisiae* (19, 20), this is predicted to trigger a conformational change within Cap1 that masks the nuclear export sequence (NES) from the Crm1 nuclear export factor, thereby allowing the nuclear accumulation of this transcription factor. Once in the nucleus, Cap1 is phosphorylated, and the induction of Cap1-dependent genes ensues (21). As many key antioxidant genes, including *CAT1* encoding catalase and *TRX1* encoding thioredoxin, are direct Cap1 targets (22), *C. albicans* cells lacking Cap1 are exquisitely sensitive to ROS (11, 23, 24) and to phagocyte-mediated killing (25, 26).

As *C. albicans* mounts a robust response to oxidative stress *in vitro* and is more resistant to ROS than many other fungi, why is this pathogen unable to survive phagocytosis in the immunocompetent host? Recently, we demonstrated that the sensitivity of *C. albicans* to oxidative stress increases dramatically if cells are simultaneously exposed to cationic stress (27). This is relevant in the context of innate immune defenses, as following phagocytosis, there is an increased flux of the K^+^ cation into the phagosome to compensate for the anionic charge that accumulates due to the high levels of O_2_^−^ generated (5). Thus, the potency of innate immune defenses against *C. albicans* can be attributed to exposure to both oxidative and cationic stresses within the phagosome. At the molecular level, this combinatorial oxidative and cationic stress-mediated synergistic killing of *C. albicans* is due to stress pathway interference (28). Specifically, Cap1 fails to accumulate in the nucleus following exposure of *C. albicans* to combinatorial oxidative and cationic stress, and thus Cap1-dependent antioxidant genes are not induced (28). Importantly, the cationic stress-mediated inhibition of oxidative stress responses appears to be of physiological relevance, as the high fungicidal activity of human neutrophils is impaired to similar extents when either the oxidative burst or the cationic flux is inhibited (28).

Here, we dissect the mechanisms underlying the combinatorial stress-mediated inhibition of Cap1 activation. We show that Cap1 becomes trapped in a partially oxidized form for sustained periods following combinatorial oxidative and cationic stress and also in response to high levels of oxidative stress. Significantly, Cap1-dependent gene expression does not occur when Cap1 is in this partially oxidized state. However, the failure of Cap1 to accumulate in the nucleus is specific to the combinatorial cationic and oxidative stress, due to the cation-mediated stabilization of the interaction between Cap1 and the Crm1 nuclear export factor. We propose that these previously uncharacterized mechanisms, which prevent the rapid activation of Cap1, underlie the exquisite sensitivity of *C. albicans* to combinatorial cationic and oxidative stress and hence the potency of innate immune defenses.

## RESULTS

### Differential oxidation of Cap1 following combinatorial stress.

**FIG 1:**
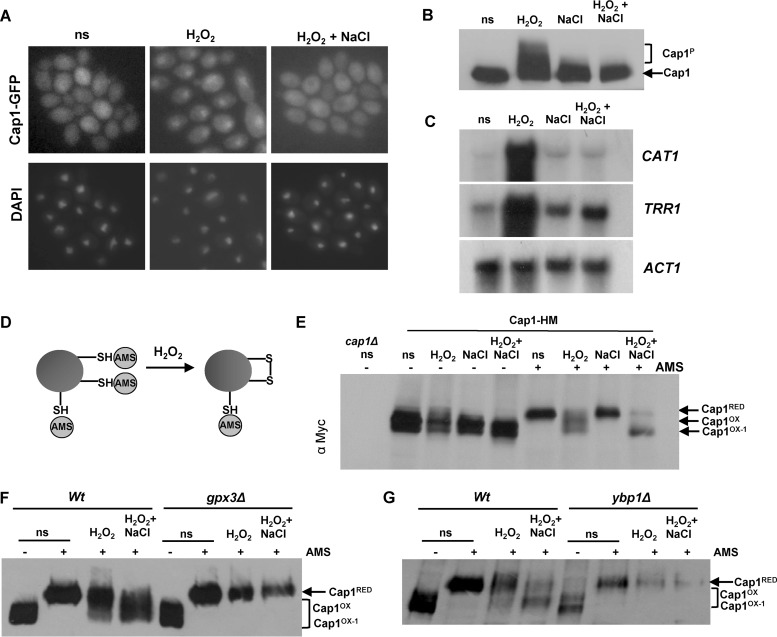
The lack of antioxidant gene expression following combinatorial stress is accompanied by a differentially oxidized form of the Cap1 transcription factor. (A) Cap1 does not accumulate in the nucleus following combinatorial stress. Localization of Cap1 was detected by fluorescence microscopy of cells expressing Cap1-GFP (JC1060) under non-stress conditions (ns) and after exposure to 5 mM H_2_O_2_ or 5 mM H_2_O_2_ plus 1 M NaCl for 10 min. The position of the nuclei is shown by DAPI staining. (B) Cap1 is phosphorylated following H_2_O_2_ exposure but not combinatorial stress. Lysates from cells expressing 2Myc- and 6His-tagged Cap1 (Cap1-MH [JC948]), before (ns) and after the indicated stress treatments, were analyzed by Western blotting using an anti-Myc antibody. The positions of nonphosphorylated (Cap1) and phosphorylated (Cap1^P^) Cap1 are indicated. (C) Combinatorial stress inhibits H_2_O_2_-induced antioxidant gene expression. Northern blot analysis of RNA isolated from wild-type (Wt [JC747]) cells before (ns) and following a 10-min treatment with 5 mM H_2_O_2_, 1 M NaCl, or 5 mM H_2_O_2_ plus 1 M NaCl. Blots were analyzed with probes specific for the catalase (*CAT1*) and thioredoxin reductase (*TRR1*) genes. A probe against *ACT1* was used as the loading control. (D) Diagram illustrating the detection of protein oxidation by AMS binding to reduced thiols. (E) Cap1 is differentially oxidized following combinatorial stress. Cap1 oxidation was analyzed by nonreducing SDS-PAGE and Western blotting of AMS-modified or untreated proteins prepared from cells expressing Cap1-HM before (ns) and following the stress treatments described above. Extracts from *cap1*Δ cells were included as a control. The positions of reduced (Cap1^RED^), oxidized (Cap1^OX^), and differentially oxidized (Cap1^OX-1^) Cap1 are indicated. (F) Differential oxidation of Cap1 is dependent on Gpx3. Cap1 oxidation was determined as described above in wild-type (Wt [JC948]) and *gpx3*Δ (JC1311) cells expressing Cap1-MH before (ns) and following the stress treatments described above. (G) Differential oxidation of Cap1 is dependent on Ypb1. Cap1 oxidation was determined as described above in wild-type (Wt [JC948]) and *ybp1*Δ (JC954) cells.

Previously we demonstrated that the normal transcriptional response to oxidative stress is not induced following the simultaneous exposure of *C. albicans* cells to cationic (1 M NaCl) and oxidative (5 mM H_2_O_2_) stress and that the major regulator of oxidative stress response gene expression, Cap1, fails to accumulate in the nucleus following such combinatorial stress treatments (28). However, the mechanisms underlying this inhibition of Cap1 function are unknown. As described above, the oxidative stress-induced nuclear accumulation of fungal AP-1-like transcription factors, such as Cap1, is triggered by the oxidation of redox-active cysteine residues. Therefore, we examined Cap1 oxidation alongside other readouts of Cap1 activation following combinatorial oxidative plus cationic stress treatments. Cells expressing Cap1 tagged with 2 copies of the Myc epitope were collected following a 10-min treatment with H_2_O_2_ or combinatorial H_2_O_2_ plus NaCl, and samples were simultaneously processed to examine Cap1 oxidation, phosphorylation, and Cap1-dependent gene expression. In addition, cells expressing Cap1-green fluorescent protein (GFP) were exposed to the same stress treatments. As expected, Cap1 failed to accumulate in the nucleus following a 10-min combinatorial H_2_O plus NaCl treatment but rapidly accumulated in the nucleus following H_2_O_2_ treatment alone (Fig. 1A). Furthermore, consistent with the effects of these stress conditions on nuclear accumulation, Cap1 was not phosphorylated following combinatorial stress treatment but was robustly phosphorylated following exposure to H_2_O_2_ alone (Fig. 1B). Analysis of the Cap1-dependent transcripts *CAT1* and *TRR1* reaffirmed our previous microarray data (28) that exposure of *C. albicans* to oxidative stress in the presence of cationic stress prevents the rapid induction of Cap1-regulated genes (Fig. 1C). As Cap1 oxidation is essential to drive the nuclear accumulation of Cap1 and Cap1-dependent gene expression, it was possible that cationic stress interferes with the H_2_O_2_-induced oxidation of Cap1. To examine this hypothesis, the redox status of Cap1 was determined using the alkylating agent 4-aceto-4′-maleimidylstilbene-2,2′-disulfonic acid (AMS) (Fig. 1D), which reacts specifically with the thiol groups of reduced cysteine residues, thereby increasing the molecular mass of thiol-modified proteins by 0.64 kDa/cysteine (21). The oxidation of cysteine residues prevents AMS binding, and consequently oxidized proteins have a lower molecular mass and faster mobility on nonreducing PAGE compared to the corresponding reduced proteins. We detected this mobility shift by Western blotting. Cells were subjected to acid lysis and reduced cysteine residues labeled with AMS. Strikingly, AMS-treated Cap1 exhibited a faster mobility following the combinatorial oxidative and cationic stress, compared to oxidative stress alone (Fig. 1E). Importantly the increased mobility of Cap1 following combinatorial stress was AMS dependent (Fig. 1E), indicating this was due to a greater number of cysteine residues being oxidized in Cap1 under these conditions, thus preventing AMS binding. We designated this differentially oxidized form “Cap1^OX-1^” and the form generated following H_2_O_2_ treatment alone “Cap1^OX^” (Fig. 1E). We also confirmed that the faster-mobility forms seen following oxidative and combinatorial stresses were not due to proteolysis. These forms were not seen in the absence of AMS following acid lysis (Fig. 1E) or following analysis of native extracts (see Fig. S1A in the supplemental material), and the cellular levels of Cap1 were not affected by any of the stress treatments employed above (see Fig. S1A and S1B).

The oxidation of *C. albicans* Cap1 in response to H_2_O_2_ requires the thiol peroxidase Gpx3 (26). Hence, we next investigated whether the formation of Cap1^OX-1^ following combinatorial cationic and oxidative stress is also dependent on Gpx3. Wild-type cells and cells lacking *GPX3* were exposed to both oxidative and combinatorial oxidative and cationic stresses, and Cap1 oxidation was examined. Interestingly, the formation of Cap1^OX-1^ following combinatorial stress, as well as Cap1^OX^ following oxidative stress, was abolished in *gpx3*Δ cells (Fig. 1F). Gpx3-mediated Cap1 oxidation also requires an orthologue of the *S. cerevisiae* Yap1 binding protein, Ybp1, which additionally functions to prevent the degradation of these AP-1-like transcription factors (26). Similar to what was observed in *gpx3*Δ cells, no Cap1 oxidation was evident in cells lacking *YBP1* following exposure to either oxidative or combinatorial stress (Fig. 1G). Furthermore, consistent with previous findings, Cap1 levels were significantly reduced in *ybp1*Δ cells (Fig. 1G). Based on these results, we conclude that differentially oxidized forms of Cap1 are generated following a 10-min exposure to oxidative and combinatorial stresses and that Gpx3 and Ybp1 are crucial for this differential Cap1 oxidation.

### Cap1^OX^ contains more disulfides than Cap1^OX-1^.

**FIG 2:**
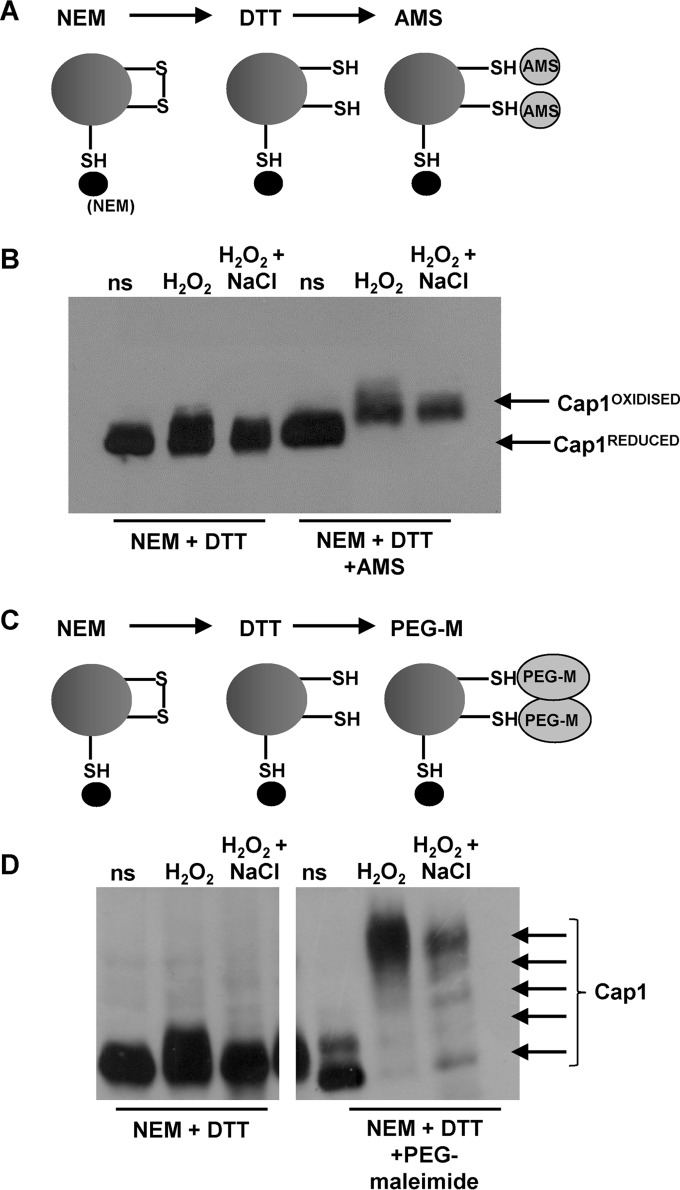
Cap1 oxidation following oxidative and combinatorial stresses. (A) Diagram showing the sequential NEM-DTT-AMS treatments employed to allow for the detection of disulfide bonds. (B) The oxidized forms generated following both oxidative and combinatorial stresses contain disulfide bonds. Cap1 mobility was monitored by nonreducing SDS-PAGE and Western blotting of proteins prepared from cells expressing Cap1-HM, under non-stress conditions (ns [Cap1^REDUCED^]) or following treatment with the indicated compounds. Samples were phosphatase treated prior to loading. The presence of disulfide bonds is indicated by the retarded mobility of Cap1 due to AMS binding to DTT-resolved disulfides (Cap1^OXIDISED^). (C) Diagram showing the sequential NEM-DTT-PEG-maleimide (PEG-M) treatments that allow for the detection of disulfide bonds. (D) Comparison of Cap1 oxidation before (ns) and following oxidative and combinatorial stresses by PEG-maleimide binding to DTT-resolved thiols. As in panel B, samples were phosphatase treated prior to loading. This shows that different oxidized forms of Cap1 are present following combinatorial stress, whereas a single form containing multiple disulfides is prevalent following oxidative stress.

In *S. cerevisiae*, the stepwise oxidation of all six redox-active cysteines within Yap1, leading to three interdomain disulfides between the n-CRD and c-CRD cysteine-rich domains, is necessary for maximal activation (29). Hence, to investigate the nature of the Cap1^OX-1^ form, labeling experiments were performed to allow for the detection of H_2_O_2_-induced disulfide bond formation. Cells were subjected to acid lysis, and free reduced cysteines were blocked with the low-molecular-mass thiol-binding reagent *N*-ethylmaleimide (NEM). Any disulfides present were then reduced with dithiothreitol (DTT), and subsequent free thiols were labeled with AMS (Fig. 2A). Thus, in this experiment, the presence of oxidized intramolecular disulfides is indicated by the reduced mobility of Cap1 upon SDS-PAGE. Cap1 exhibited reduced mobility after the sequential NEM-DTT-AMS treatment in cells treated with either oxidative stress or combinatorial oxidative plus cationic stress (Fig. 2B; see Fig. S2A in the supplemental material). Thus, both Cap1^OX^ and Cap1^OX-1^ forms contain oxidized intramolecular disulfides. Based on our previous experiment, in which Cap^OX-1^ displayed a faster mobility than Cap1^OX^ following AMS treatment alone (Fig. 1E), we predicted that this form of Cap1 may have more disulfides than Cap1^OX^. If this was the case, then following sequential NEM-DTT-AMS treatment, Cap1^OX-1^ would have a slower mobility than Cap1^OX^ due to more AMS binding. However, this was not observed: Cap1 displayed a slightly slower mobility following oxidative stress than following combinatorial stress (Fig. 2B). This suggested that Cap1^OX-1^ has fewer disulfides than Cap1^OX^. It was possible, however, that this slower mobility of Cap1 following oxidative stress was due to residual phosphorylation of this transcription factor, even after phosphatase treatment, as phosphorylation occurs following H_2_O_2_, but not combinatorial, stress treatments (Fig. 1C). To avoid this complication, we repeated the experiment by monitoring mobility retardation mediated by another thiol-alkylation probe, polyethylene glycol (PEG)-linked maleimide, which has a higher molecular mass (2 kDa) than AMS (Fig. 2C). Using PEG-maleimide, clear differences were observed in the oxidized forms of Cap1 generated following oxidative and combinatorial stress treatment (Fig. 2D). Following H_2_O_2_ stress, Cap1 was present predominantly as a single species with significantly retarded mobility following the sequential NEM-DTT-PEG maleimide treatment. In contrast, following combinatorial stress, multiple differentially oxidized forms of Cap1 were observed, with only a fraction displaying the same retarded mobility as that seen for Cap1^OX^ following oxidative stress alone (Fig. 2D). Thus, although the previous AMS binding experiment indicated that Cap1^OX-1^ was more oxidized than Cap1^OX^ (Fig. 1E), these experiments indicate that Cap1^OX^ has more H_2_O_2_-induced disulfides than Cap1^OX-1^, generated following combinatorial stress. This seemingly contradictory observation could be explained by the hyperoxidation of cysteine thiols to sulfinic or sulfonic acid derivatives in the Cap1^OX-1^ form.

### Differential oxidation and inactivation of Cap1 following combinatorial stress is transient.

**FIG 3:**
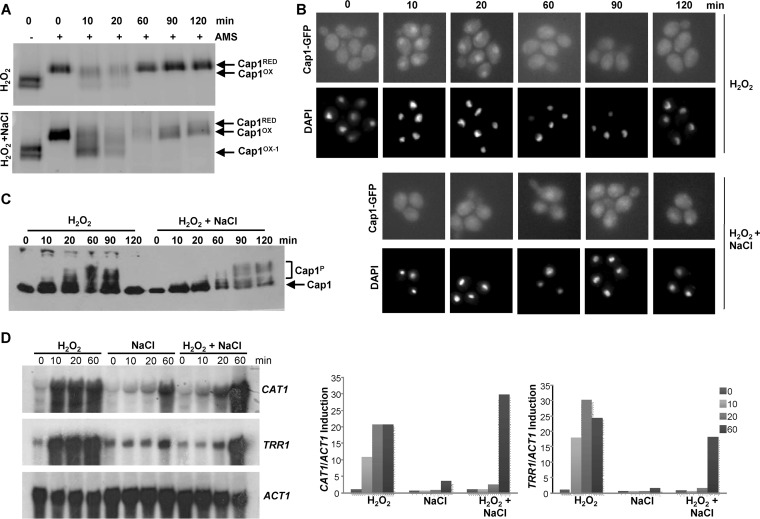
Combinatorial stress-mediated inhibition of Cap1 activation is transient. (A) Differential oxidation of Cap1 following combinatorial stress is not sustained. Cap1 oxidation was measured as described in the legend to Fig. 1E following exposure of Cap1-MH cells to 5 mM H_2_O_2_ or 5 mM H_2_O_2_ plus 1 M NaCl for the indicated times. (B) Cap1 nuclear accumulation is delayed following combinatorial stress. Cap1 localization was detected as described in the legend to Fig. 1B following treatment of Cap1-GFP cells for the indicated times with the stress treatments described above. (C) Cap1 phosphorylation is delayed following combinatorial stress treatment. Cap1 phosphorylation was detected as described in the legend to Fig. 1C, following treatment of Cap1-MH cells with the stress treatments described above for the indicated times. (D) The inhibition of Cap-dependent gene expression following combinatorial stress is transient. Northern blots were performed as described in the legend to Fig. 1A, after wild-type cells were treated with the stress treatments described above for the indicated times (left panel). The levels of *CAT1* and *TRR1* mRNA were quantified relative to the *ACT1* loading control (right panel).

Next, we determined whether the differential oxidation and inactivation of Cap1 following combinatorial stress was short-lived or irreversible. Cells expressing either Myc-tagged Cap1 or Cap1-GFP were treated with H_2_O_2_ or H_2_O_2_ plus NaCl, and samples were collected over a 2-h period. First of all, we determined the redox status of Cap1 using the AMS alkylating agent as described in the legend to Fig. 1D. The double band of Cap1 seen in the non-AMS-treated time zero sample is likely due to oxidation during protein extraction as this is prevented by the addition of the low-molecular-mass thiol binding agent NEM during extraction (see Fig. S2B in the supplemental material). Consistent with previous findings, the Cap1^OX^ form was quickly generated following H_2_O_2_ stress and then resolved back to the reduced form within 60 min. Similarly, the Cap1^OX-1^ form appeared rapidly after H_2_O_2_ plus NaCl stress (Fig. 3A). However, by 60 min, the combinatorial stress-induced Cap1^OX-1^ form was resolved to a form with mobility similar to that of Cap1^OX^ (Fig. 3A). This indicates that the Cap1^OX-1^ form generated following combinatorial stress is transient.

To examine whether the inactivation of Cap1 following combinatorial stress was coincident with the presence of the Cap1^OX-1^ form, the kinetics of Cap1 nuclear accumulation, phosphorylation, and Cap1-dependent gene expression were determined. As illustrated in Fig. 3B, Cap1 did accumulate in the nucleus following combinatorial H_2_O_2_ plus NaCl stress, but with significantly delayed kinetics compared to H_2_O_2_ stress. Cap1 located to the nucleus 10 min following H_2_O_2_ stress, whereas nuclear accumulation of Cap1 post-combinatorial H_2_O_2_ and NaCl stress was not evident until 60 min. Notably the appearance of Cap1 in the nucleus coincided with Cap1 being resolved to the Cap1^OX^ form (Fig. 3A and B). We next examined Cap1 phosphorylation, as this posttranslational modification is associated with the nuclear accumulation of fungal AP-1-like transcription factors (30). Cap1 phosphorylation was only seen 60 min after the combinatorial H_2_O_2_ plus NaCl stress, coincident with the point at which Cap1 accumulated in the nucleus (Fig. 3C). Significantly, the delayed nuclear accumulation and phosphorylation of Cap1 observed following combinatorial stress was mirrored by a delay in Cap1-dependent gene induction (Fig. 3D). Northern analysis revealed that the Cap1-dependent genes *CAT1* and *TRR1* are significantly induced following the combinatorial H_2_O_2_ plus NaCl stress, but not until 60 min after the combinatorial stress treatment. In contrast, these key antioxidant-encoding genes were induced within 10 min of H_2_O_2_ stress exposure (Fig. 3D). This is entirely consistent with our previous microarray data which failed to detect Cap1-dependent gene expression following a 10-min exposure to the combinatorial oxidative plus cationic stress (28). The significant delay in Cap1-dependent gene expression following simultaneous exposure to H_2_O_2_ plus NaCl likely underlies the inability of *C. albicans* to survive this combination of stresses.

### Differential Cap1 oxidation triggered by high levels of H_2_O_2_.

**FIG 4:**
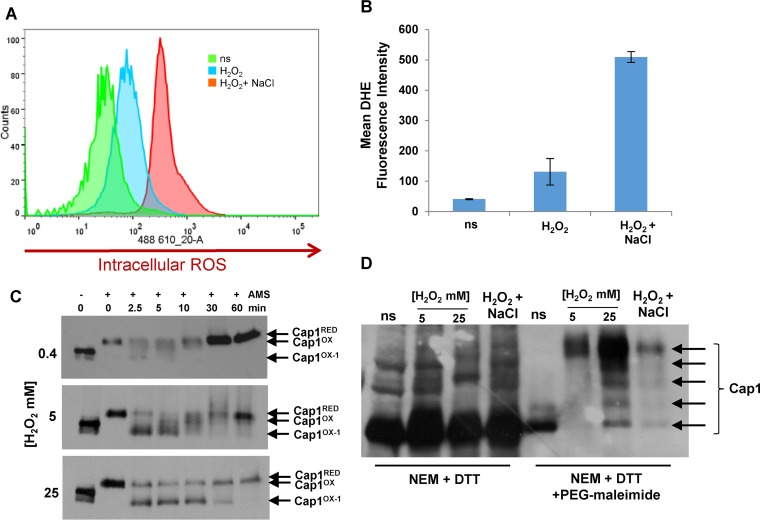
Combinatorial stress induces high levels of intracellular ROS, and high levels of H_2_O_2_ also result in sustained differential Cap1 oxidation. (A) Fluorescence-activated cell sorter (FACS) analysis of intracellular ROS levels in DHE-treated *C. albicans* cells before stress (ns) or following the treatment with 5 mM H_2_O_2_ or 5 mM H_2_O_2_ plus 1 M NaCl for 60 min. (B) Quantification of intracellular ROS production before (ns) and following treatment with the indicated stresses by calculating the mean DHE fluorescence intensity of the area under the curve. The mean ± standard deviation (SD) from three independent experiments is shown. (C) Cap1^OX-1^ is sustained following treatment with higher doses of H_2_O_2_. Cap1 oxidation was measured as described in the legend to Fig. 1D following exposure of Cap1-MH cells to 0.4, 5, or 25 mM H_2_O_2_ for the times indicated. (D) Comparison of Cap1 oxidation before (ns) and following the indicated stress treatments by PEG-maleimide binding to DTT-resolved thiols. This shows that different oxidized forms of Cap1 are similarly triggered following high-H_2_O_2_ and combinatorial stresses, whereas a single form containing multiple disulfides is prevalent following medium-H_2_O_2_ stress.

Why does Cap1 become differentially oxidized following combinatorial H_2_O_2_ and NaCl stress? We previously reported that there is a dramatic increase in intracellular ROS levels following exposure to combinatorial oxidative plus cationic stress, compared to oxidative stress alone (28). This rise in intracellular ROS could drive the differential oxidation and inactivation of Cap1. To explore this, we first quantified the increase in intracellular ROS following treatment of cells with H_2_O_2_ or combinations of H_2_O_2_ and NaCl. Approximately 5-fold-higher levels of intracellular ROS were observed following exposure of cells to 5 mM H_2_O_2_ in the presence of 1 M NaCl, compared to 5 mM H_2_O_2_ alone (Fig. 4A and B). Based on this observation, we hypothesized that Cap1 may also become differentially oxidized to the Cap1^OX-1^ form following exposure of cells to high levels of ROS. To investigate this, cells expressing Myc-tagged Cap1 were treated with low (0.4 mM), medium (5 mM), and high (25 mM) levels of H_2_O_2_, and Cap1 oxidation was monitored over a 60-min period. Strikingly, this kinetic analysis revealed that, at all levels of H_2_O_2_ tested, a faster-mobility Cap1^OX-1^ form is rapidly observed after stress treatment. However, following exposure to low or medium levels of H_2_O_2_, the presence of Cap1^OX-1^ is short-lived, whereas following exposure to high levels of H_2_O_2_, Cap1^OX-1^ persists for up to 30 min (Fig. 4C). Significantly, as a Cap1^OX-1^ form is seen at all H_2_O_2_ concentrations, albeit with different kinetics, this indicates that such a form may in fact be a normal intermediate in the formation of the Cap1^OX^ form following H_2_O_2_ stress.

We explored whether the Cap1^OX-1^ form, generated for a sustained period following high levels of H_2_O_2_, was similar to the Cap1^OX-1^ form observed after combinatorial cationic and oxidative stress, using the sequential NEM-DTT-PEG maleimide treatments described above. As observed previously (Fig. 2D), exposure of cells to medium (5 mM) doses of H_2_O_2_ results in a single species of Cap1 predicted to contain multiple disulfide bonds, as evidenced by the slow, PEG-maleimide-dependent mobility on SDS-PAGE (Fig. 4D). In contrast, treatment of cells with either high (25 mM) doses of H_2_O_2_ or combinatorial stress results in the formation of multiple different oxidized forms of Cap1. For unknown reasons, we consistently recovered more Cap1 following high-H_2_O_2_ stress than other treatments (Fig. 4D). However, it is clear that the same differentially oxidized forms of Cap1 are observed following either high-H_2_O_2_ or combinatorial stress (Fig. 4D).

**FIG 5:**
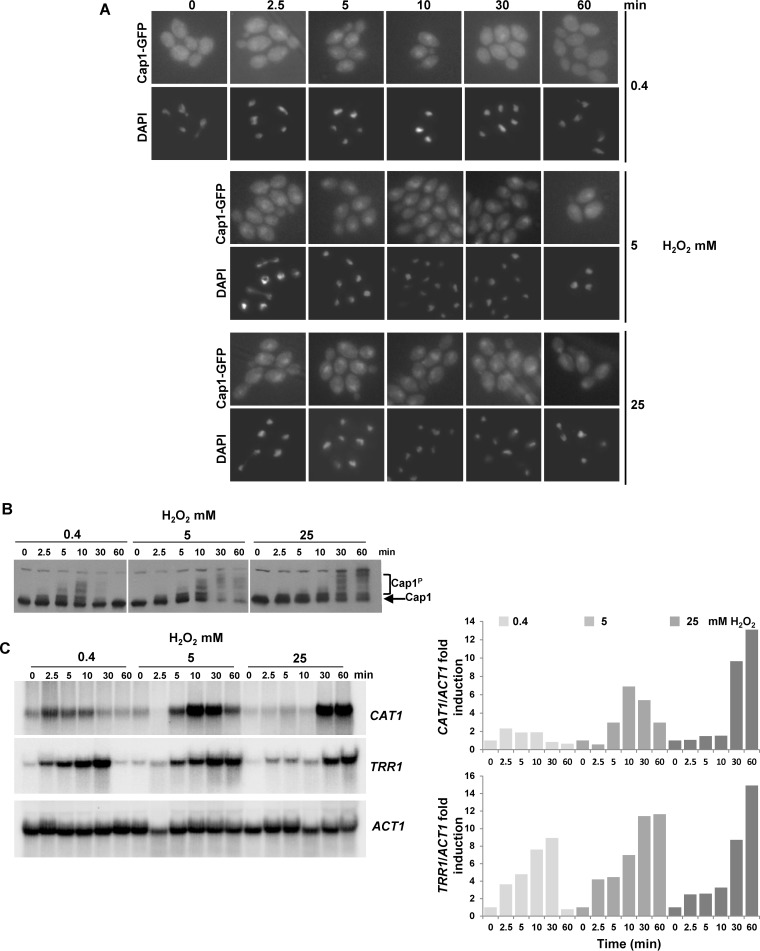
Cap1 rapidly accumulates in the nucleus in response to high levels of H_2_O_2_, but Cap1 phosphorylation and Cap1-dependent gene expression are delayed. (A) Cap1 localization was detected as described in the legend to Fig. 1B following treatment of Cap1-GFP cells with 0.4, 5, or 25 mM H_2_O_2_ for the times indicated. (B) Cap1 phosphorylation was detected as described in the legend to Fig. 1C following treatment of wild-type cells with 0.4, 5, or 25 mM H_2_O_2_ for the times indicated. (C) Northern blots were performed as described in the legend to Fig. 1A, using the same cells that were processed for Cap1 phosphorylation analysis in panel B (left panel). The levels of *CAT1* and *TRR1* mRNA were quantified relative to the *ACT1* loading control (right panel).

As Cap1-dependent gene expression is delayed following combinatorial oxidative and osmotic stress when the Cap1^OX-1^ form is prevalent (Fig. 3D), we hypothesized that the delayed conversion of Cap1^OX-1^ to Cap1^OX^ following higher levels of H_2_O_2_ (Fig. 4C) may also result in an H_2_O_2_ concentration-dependent lag in Cap1-dependent gene expression. To investigate this, we examined the kinetics of Cap1 nuclear accumulation, phosphorylation, and Cap1-dependent gene expression following the exposure of cells to low (0.4 mM), medium (5 mM), and high (25 mM) doses of H_2_O_2_. The first observation made was that, in contrast to combinatorial H_2_O_2_ and NaCl stress, Cap1 rapidly accumulated in the nucleus irrespective of the level of H_2_O_2_ stress (Fig. 5A). Following exposure of cells to 0.4 mM H_2_O_2_, this nuclear accumulation is short-lived, consistent with the transient oxidation of Cap1 (Fig. 4C). However, Cap1 nuclear accumulation persists for 60 min following treatment with either 5 or 25 mM H_2_O_2_ (Fig. 5A). Thus, while the Cap1^OX-1^ form generated following combinatorial stress fails to accumulate in the nucleus, Cap1^OX-1^ formed in response to high levels of H_2_O_2_ rapidly accumulates in the nucleus (compare Fig. 5A with Fig. 3B).

Strikingly, however, despite the fast nuclear accumulation following high levels of H_2_O_2_, Cap1 phosphorylation (Fig. 5B) and Cap1-dependent gene expression (Fig. 5C) were not observed until 30 min post-stress treatment. The delayed phosphorylation of Cap1 appeared to coincide with the resolution of the Cap1^OX-1^ form to the Cap1^OX^ form (Fig. 4C), which in turn correlated with the timing of Cap1-dependent gene expression. Such observations are indicative of a connection between these Cap1 modifications and the activity of this transcription factor. In contrast, consistent with previous findings (31), following low and medium doses of H_2_O_2_, the kinetics of induction of Cap1-dependent genes (Fig. 5C) largely correlated with the oxidation, phosphorylation, and nuclear accumulation profiles of Cap1 (Fig. 4C and Fig. 5A and B).

**FIG 6:**
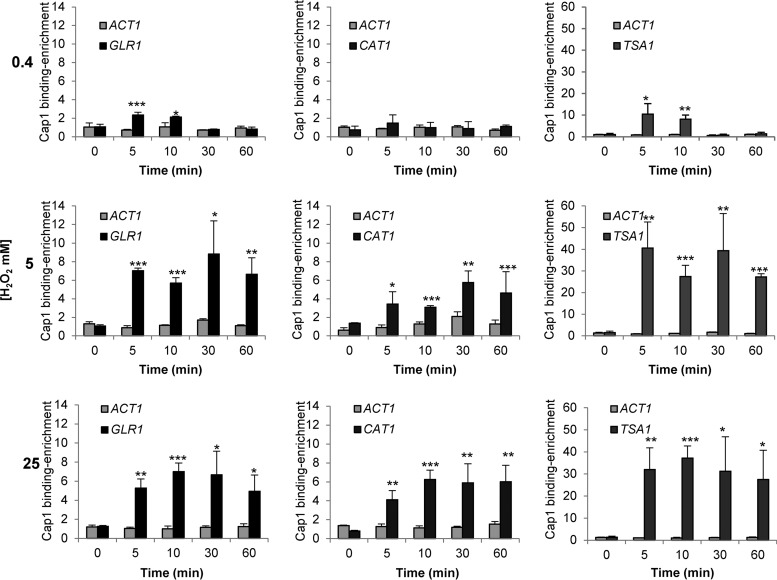
Quantification of Cap1 enrichment at the *GLR1*, *CAT1*, and *TSA1* promoter targets. Cells expressing Cap1-MH (JC948) were treated with 0.4, 5, or 25 mM H_2_O_2_ for the indicated times and subjected to ChIP. The recovered DNA samples were analyzed by qPCR using primers specific for promoter regions within *GLR1*, *CAT1*, and *TSA1.* Relative Cap1-MH enrichment values (*n*-fold) are presented (mean ± SD; *n* = 3). The following *P* values were considered: *, *P* ≤ 0.05; **, *P* ≤ 0.01; and ***, *P* ≤ 0.001.

Cap1-dependent gene expression is delayed following high levels of H_2_O_2_, despite the clear nuclear accumulation of Cap1. Therefore, we explored whether Cap1 was bound to its target genes under such conditions. To examine Cap1 promoter binding, three targets were selected—*CAT1*, *GLR1*, and *TSA1*—based upon previous studies showing Cap1 enrichment at their promoters (22) and the Cap1-dependent induction of such genes in response to 5 mM H_2_O_2_ (11, 16, 32). *C. albicans* cells expressing Myc-tagged Cap1 together with an untagged control strain were treated with the low, medium, and high levels of H_2_O_2_ employed above, and Cap1 binding to the *CAT1*, *GLR1*, and *TSA1* promoters was determined by chromatin immunoprecipitation (ChIP) and quantitative PCR analysis. As illustrated in Fig. 6, Cap1 was found to rapidly associate with *CAT1*, *GLR1*, and *TSA1* promoters irrespective of the level of H_2_O_2_ (Fig. 6). Moreover, considerably higher levels of Cap1 were detected at these promoters following treatment with medium or high levels H_2_O_2_ compared to low doses (Fig. 6). Clearly therefore, the unphosphorylated Cap1^OX-1^ form generated following high levels of H_2_O_2_ is able to bind to the promoter region of its target genes. However, in contrast to the effect seen at low and medium levels of H_2_O_2_, this is not sufficient to drive Cap1-dependent antioxidant gene expression (Fig. 5C).

Taken together, these results highlight a number of significant findings regarding Cap1 regulation in *C. albicans*. First, it is apparent that Cap1-dependent gene expression is delayed following exposure of cells to high levels of H_2_O_2_. Importantly, this may underlie the delayed Cap1-dependent gene expression seen following the combinatorial H_2_O_2_ and NaCl stress, as this combination of stresses triggers high levels of intracellular ROS. Second, the nuclear Cap1^OX-1^ form, generated following high levels of oxidative stress, although bound to the promoters of Cap1-regulated genes, is not phosphorylated and fails to activate its target genes. These findings suggest that the resolution of the Cap1^OX-1^ form to Cap1^OX^ and Cap1 phosphorylation are necessary prerequisites for the induction of the Cap1-mediated oxidative stress regulon.

### Cationic stress promotes the interaction of Cap1 with the Crm1 nuclear export factor.

**FIG 7:**
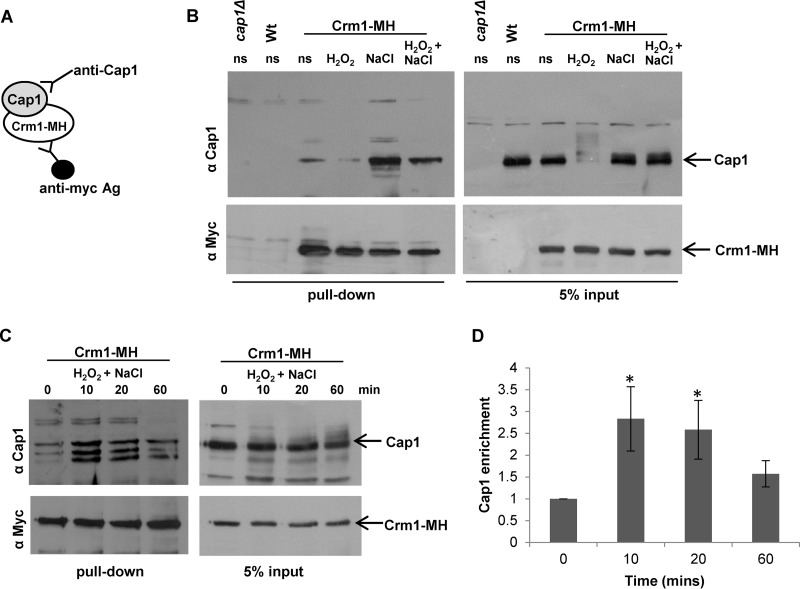
Cationic stress promotes Cap1 interaction with the Crm1 nuclear export factor. (A) Strategy used to examine Cap1 association with Crm1. (B) Stress effects on Cap1 interaction with Crm1. Extracts were prepared from wild-type cells (Wt [JC747]), *cap1*Δ cells (JC842), and wild-type cells expressing 2Myc- and 6His-tagged Crm1 (Crm1-MH [JC1925]) before (ns) and following exposure to 5 mM H_2_O_2_, 1 M NaCl, or combinations of the stresses for 10 min. Crm1-MH was immunoprecipitated using anti-Myc agarose, and precipitated proteins and 5% input were subjected to SDS-PAGE. Coprecipitation of Cap1 was assayed by Western blotting using an anti-Cap1 antibody (top panel), and precipitation of Crm1-MH was assayed using anti-Myc antibodies (bottom panel). (C) The increased interaction of Cap1 with Crm1 following combinatorial stress is transient. Cap1 interaction with Crm1 was analyzed as described for panel B before (ns) and following treatment of Crm1-MH cells with 5 mM H_2_O_2_ plus 1 M NaCl for the times indicated. (D) Quantification of the increased interaction of Cap1 with Crm1 following combinatorial stress. Quantitative densitometric analysis of Western blots from four biological replicates was conducted to determine the fold enrichment of Cap1 interaction with Crm1 relative to time zero. Mean values (±standard errors of the mean [SEM]) are shown, and ANOVA was used to determine statistically significant differences in Cap1 enrichment levels (*, *P* < 0.01).

The results presented above illustrate that exposure of cells to either high levels of H_2_O_2_ (25 mM) or medium doses of H_2_O_2_ (5 mM) in the presence of NaCl results in the sustained formation of Cap1^OX-1^ and a delay in the activation of Cap1-dependent gene expression. However, while Cap1^OX-1^ rapidly accumulates in the nucleus in response to high levels of H_2_O_2_ (Fig. 5A), Cap1^OX-1^ does not accumulate in the nucleus until 1 h following combinatorial H_2_O_2_ and NaCl treatment (Fig. 3B). The H_2_O_2_-induced nuclear accumulation of fungal AP-1-like transcription factors is triggered by the oxidation of key cysteine residues, which results in a conformational change that masks the NES from the nuclear export factor Crm1 (20). Thus, the Cap1^OX-1^ forms generated following high-level H_2_O_2_ stress and combinatorial stress could conceivably be different, with the combinatorial stress Cap1^OX-1^ form adopting a structural conformation that still permits interaction with the Crm1 nuclear export factor. However, our data indicate that the oxidation profiles of Cap1^OX-1^ observed following high-H_2_O_2_ and combinatorial stresses are similar (Fig. 4D). Alternatively, it was possible that cationic stress modulates the interaction between Cap1 with the Crm1 nuclear export factor and thus affects the nuclear accumulation of Cap1^OX-1^. To test the latter hypothesis, we first created strains in which Crm1 tagged with 6His residues and 2Myc epitopes (Crm1-MH) was expressed from its native chromosomal locus. Crm1-MH was immunoprecipitated from extracts prepared from cells before and after exposure to H_2_O_2_, NaCl, and H_2_O_2_ plus NaCl. The interaction between Cap1 and Crm1 was then examined by Western blot analysis of these coimmunoprecipitates (Fig. 7A; see Fig. S3A in the supplemental material). Similar to previous studies of Yap1 in *S. cerevisiae* (33), Cap1 was found to interact with Crm1 *in vivo*, and moreover, this interaction was reduced in the presence of medium (5 mM) levels of H_2_O_2_ after 10 min (Fig. 7B; see Fig. S3A). Strikingly, the interaction of Cap1 with Crm1 was dramatically enhanced following the cationic stress (NaCl) and the combinatorial H_2_O_2_ and NaCl stress treatments (Fig. 7B; see Fig. S3A). Input controls confirmed that the cationic stress-induced interaction with Crm1 was not due to differences in Cap1 levels (Fig. 7B). The input controls, in contrast to the immunoprecipitated samples, were not phosphatase treated, which explains the slower mobility of Cap1 following oxidative stress seen in the input panel (Fig. 7B). The enhanced interaction between Cap1 and Crm1 following the combinatorial H_2_O_2_ and NaCl stress was transient, declining 1 h post-stress treatment (Fig. 7C). Quantification of the interaction of Cap1 with Crm1 from four biological replicates indicated a significant increase in Cap1 binding 10 and 20 min after combinatorial stress, but not after 60 min (Fig. 7D). This is consistent with the timing of Cap1 nuclear accumulation, which is observed 1 h following combinatorial H_2_O_2_ and NaCl stress (Fig. 3B). However, it is noteworthy that multiple species of Cap1 were often detected during the immunoprecipitation experiment (Fig. 7C; see Fig. S3A). These may reflect degradation products generated during the immunoprecipitation procedure. Taken together, these results support a model in which Cap1^OX-1^ fails to accumulate in the nucleus following exposure of cells to H_2_O_2_ in the presence of NaCl, due to the cation-enhanced interaction between Cap1 and the Crm1 nuclear export factor.

## DISCUSSION

**FIG 8:**
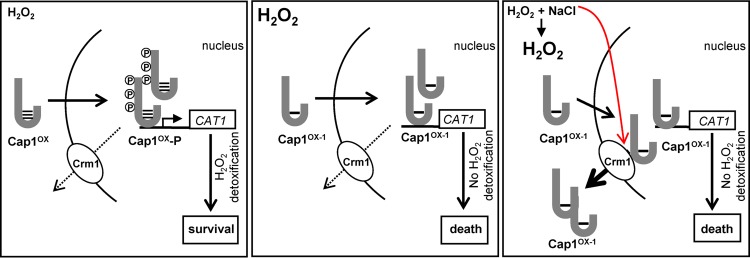
Impact of combinatorial stress and high H_2_O_2_ on Cap1 activation. When *C. albicans* cells are exposed to medium (5 mM) levels of H_2_O_2_, a Cap1^OX^ form containing multiple disulfides is swiftly generated, followed by the rapid accumulation of Cap1 in the nucleus, where it is phosphorylated. The ensuing efficient induction of Cap1-dependent antioxidant genes follows, leading to stress adaptation and survival (left panel). However, when *C. albicans* cells are exposed to high (25 mM) levels of H_2_O_2_, a Cap1^OX-1^ form containing less disulfides is prevalent, and although this rapidly accumulates in the nucleus and binds to target genes, Cap1 is not phosphorylated, and the induction of Cap1-dependent genes is significantly impaired, resulting in cell death (middle panel). Exposure of *C. albicans* to medium (5 mM) levels of H_2_O_2_ in the presence of NaCl triggers high intracellular ROS accumulation. This also results in the formation of Cap1^OX-1^, which is seemingly unable to induce Cap1-dependent genes. However, following combinatorial stress, an additional inhibitory mechanism is seen as Cap1 fails to accumulate in the nucleus due to a cationic stress-mediated stabilization of the interaction between Cap1 and the Crm1 export factor. Consequently, the induction of Cap1 target genes is prevented, leading to cell death (right panel).

In this study, we have demonstrated that exposure of *C. albicans* cells to combinatorial H_2_O_2_ plus NaCl stress, which triggers high levels of intracellular ROS, results in the sustained formation of a differentially oxidized form of Cap1, Cap1^OX-1^. Furthermore, we show that Cap1^OX-1^ contains less oxidized disulfide bonds compared to those seen in the active Cap1^OX^ form. This differentially oxidized Cap1^OX-1^ form is also observed following treatment of *C. albicans* cells with a range of H_2_O_2_ doses but is only sustained following high doses of H_2_O_2_ (Fig. 4). Notably, Cap1-dependent gene expression is not induced when Cap1 is in the Cap1^OX-1^ form. However, while Cap1^OX-1^ readily accumulates in the nucleus following high-H_2_O_2_ stress, the nuclear accumulation of Cap1^OX-1^ following combinatorial H_2_O_2_ and NaCl stress is delayed due to an enhanced interaction with the Crm1 nuclear export factor. Thus, at least two distinct mechanisms exist to inhibit Cap1 activation following combinatorial stress. This may explain why Cap1 inhibition is more sustained following combinatorial stress than high-H_2_O_2_ stress. A model comparing the mechanisms underlying the delayed activation of Cap1 following combinatorial H_2_O_2_ plus NaCl stress and high-H_2_O_2_ stress is shown in Fig. 8. We predict that the significant delay in Cap1-dependent gene expression following exposure of cells to H_2_O_2_ in the presence of NaCl underlies the exquisite sensitivity of *C. albicans* to this combinatorial stress. This is supported by the observation that ectopic expression of the Cap1-target gene, *CAT1*, can restore *C. albicans* resistance to combinatorial H_2_O_2_ and NaCl stress (28). In addition, our findings reported here that Cap1-dependent gene expression is also delayed following high-level H_2_O_2_ stress, albeit not to the same extent as following combinatorial stress, likely contributes to the increased sensitivity of *C. albicans* to high doses of oxidative stress. Moreover, the failure to respond rapidly to high intracellular ROS likely results in the activation of cell death programs (34).

How is Cap1 activated in *C. albicans*? Genetic and biochemical studies in *S. cerevisiae* support the stepwise oxidation of multiple redox-active cysteine residues in Yap1 following H_2_O_2_ exposure. This is initiated by the H_2_O_2_-induced oxidation of the catalytic cysteine (Cys36) of the thiol-peroxidase Gpx3, which subsequently reacts with Cys598 located within the c-CRD of Yap1 to form a mixed Yap1-Gpx3 disulfide intermediate. This is followed by thiol-disulfide exchange with Cys303 of Yap1 to generate the first interdomain disulfide bond between Cys303 and Cys598 (21). This disulfide is sufficient to mask the NES within the c-CRD of Yap1, prevent interaction with the Crm1 nuclear export factor, and thus stimulate nuclear accumulation (30). However, it is clear that disulfides in addition to Cys303 and Cys598 are required for optimal Yap1 function. Mass spectrometry analysis of oxidized Yap1 identified Cys303-Cys598 and Cys310-Cys629 disulfides (35), both of which are required to recruit the mediator component Rox3 to the *TRX2* promoter (36). Furthermore, an *in vitro* analysis of Yap1 oxidation identified a third disulfide, Cys315-Cys620, which appears to sustain Yap1 activation following H_2_O_2_ exposure (29). Thus, the stepwise oxidation of all six redox-active cysteines is necessary for maximal Yap1 activation in *S. cerevisiae*. In this study, we present evidence for a similar stepwise formation of multiple disulfides in *C. albicans* Cap1. The Cap1^OX-1^ form would appear to be a normal intermediate in the formation of active Cap1^OX^, and our experiments, in which we examine the binding of PEG-maleimide to DTT-resolved disulfides (Fig. 4D), show that Cap1^OX^ has more disulfides than Cap1^OX-1^. This result, however, seemingly conflicts with the oxidation profiles of Cap1 observed when we use the thiol binding agent AMS to detect differentially oxidized forms of this transcription factor. In such experiments, oxidation of cysteine residues precludes the binding of AMS, and thus oxidized proteins run with a faster mobility*.* Employing such an approach, we observe a faster-mobility form generated immediately following H_2_O_2_ stress (Cap1^OX-1^), which is then resolved into the Cap1^OX^ slower-mobility state (Fig. 4C). How can these findings be reconciled with our observations that Cap1^OX^ has more H_2_O_2_-induced disulfides than Cap1^OX-1^? Oxidation events, in addition to disulfide bond formation, such as the hyperoxidation of cysteine thiols to sulfinic or sulfonic acid derivatives, would preclude AMS binding. Alternatively, AMS binding to the Cap^OX-1^ form may induce a conformation change that results in the observed faster mobility. Further investigations are required to distinguish between these hypotheses and identify the precise oxidation states of the Cap1^OX^ and Cap1^OX-1^ forms in *C. albicans*.

The sustained formation of Cap1^OX-1^ following high-H_2_O_2_ and combinatorial stresses in *C. albicans* correlates with a lack of Cap1-dependent gene expression. Why is Cap1-dependent gene expression inhibited when Cap1 is in the Cap1^OX-1^ form? Following combinatorial oxidative plus cationic stress, Cap1^OX-1^ fails to accumulate in the nucleus, which would explain the lack of induction of Cap1-dependent genes. However, following high levels of H_2_O_2_, Cap1^OX-1^ rapidly accumulates in the nucleus, where it binds to the promoters of its target genes (Fig. 6), and yet still, Cap1-dependent gene induction is not observed (Fig. 5C). Here it is interesting to note that although Cap1^OX-1^ accumulates in the nucleus, it is not phosphorylated. Studies of *S. cerevisiae* revealed that the nuclear accumulation of Yap1 was necessary for this posttranslational modification (30). However, as both the kinase and target phosphorylation sites on Yap1 are unknown, it has not been possible to establish whether this posttranslational modification is important for the activation of this or other fungal AP-1-like transcription factors. Results from this study which show that there is a coordinated delay in Cap1 phosphorylation and Cap1-dependent gene expression following high-H_2_O_2_ stress, despite the nuclear accumulation of Cap1, are consistent with a model in which Cap1 phosphorylation is important for function. In this scenario, either the kinase responsible is inactivated following high-H_2_O_2_ stress, or it fails to recognize the Cap1^OX-1^ form. Indeed, Cap1 phosphorylation coincides with the appearance of the active Cap1^OX^ form. However, other, phosphorylation-independent, mechanisms may also contribute to the delay in Cap1-induced gene expression. For example, in *S. cerevisiae*, the correct oxidation of Yap1 is necessary to recruit the polymerase II (Pol II) mediator subunit Rox3 (36). Thus, the Cap1^OX-1^ form may not be competent to recruit the general transcriptional machinery. Alternatively, high levels of intracellular ROS, generated following combinatorial and high-H_2_O_2_ stresses, may have a global inhibitory effect on transcription. Indeed, high levels of ROS have been previously demonstrated to result in a global inhibition of protein translation in *S. cerevisiae* (37). To explore whether a global inhibition of transcription occurs following combinatorial and high-H_2_O_2_ stresses, we examined the phosphorylation status of RNA Pol II. Phosphorylation of serine 2 and 5 within the repetitive YSPTSPS sequence, located in the carboxyl-terminal domain (CTD), is associated with transcriptionally active RNA Pol II (38). No obvious change in RNA Pol II phosphorylation status was seen following combinatorial and high-H_2_O_2_ stresses, suggesting that under such conditions, the polymerase is active (see Fig. S4 in the supplemental material). However, additional experiments are needed to further explore the potential global impacts of these stress treatments on gene expression.

The inhibition of Cap1 nuclear accumulation following combinatorial stress represents a clear difference in Cap1 regulation following high-H_2_O_2_ stress and combinatorial cationic and oxidative stress. Strikingly, in this study we find that cationic stress appears to enhance the interaction between Cap1 and Crm1 in *C. albicans*, and this interaction is maintained following combinatorial cationic and oxidative stress treatments (Fig. 7B; see Fig. S3A in the supplemental material). Structural analysis of Yap1 revealed that in the active oxidized form, the nuclear export signal (NES) in the c-CRD is masked by disulfide-bond-mediated interactions with a conserved amino-terminal α-helix. Upon reduction of the disulfide bonds, Yap1 undergoes a change to an unstructured conformation that exposes the NES and allows redistribution to the cytoplasm (20). As cationic stress alone can promote the interaction of Cap1 with Crm1, this indicates that this phenomenon occurs independently of changes in the oxidation status of Cap1. We reasoned that the production of osmolytes, triggered by cationic stress, may be responsible for the enhanced interaction between Cap1 and Crm1. However, the cation-induced interaction between Cap1 and Crm1 is maintained in cells lacking the Hog1 stress-activated protein kinase (SAPK) (see Fig. S3B), which fail to produce glycerol following osmotic stress (39). Furthermore, the enhanced interaction appears to be cation specific, as treatment of cells with the osmotic stress agent sorbitol fails to stimulate Cap1 interaction with Crm1 (see Fig. S3C). Although the molecular basis underlying the cation-stimulated Cap1-Crm1 association is unknown, this observation can account for the significant delay in Cap1 nuclear accumulation following combinatorial oxidative and osmotic stress. Interestingly, it is possible that the cationic stress-stimulated interaction with Crm1 may be Cap1 specific, rather than a global phenomenon. For example, we have previously shown that the Hog1 SAPK, whose cellular localization is predicted to regulated by Crm1 (40), rapidly accumulates in the nucleus following combinatorial stress (28). Further investigation into the mechanisms underlying the cationic-stress mediated association between Cap1 and Crm1 is clearly warranted. Although seemingly Hog1 dependent, the Rim101 pathway is worthy of consideration as this pathway is implicated in cationic but not osmotic stress. *C. albicans* cells lacking the Rim101 transcription factor are sensitive to cationic stress (41), and a role of Rim101 in regulating the Ena1 Na^+^-ATPase in the model yeast *S. cerevisiae* is well documented (42).

In conclusion, our results provide significant new insight into why combinatorial cationic plus oxidative stress treatments delay the activation of the Cap1 transcription factor, leading to stress pathway interference. Indeed, cationic stress appears to inhibit oxidative stress responses in two ways. First, cations inhibit catalase activity in *C. albicans*, thereby leading to significant increases in intracellular ROS levels observed following combinations of cationic and oxidative stresses (28). Here we show that such high levels of intracellular ROS trap Cap1 in an intermediate Cap1^OX-1^ form that fails to induce target antioxidant-encoding genes. Second, we find that cations stimulate the interaction of Cap1 with the nuclear export factor Crm1, thus delaying the oxidative stress-induced nuclear accumulation of this transcription factor. This combinatorial stress-mediated stress pathway interference that we have dissected *in vitro* is thought to contribute to the potency of host defenses in combating fungal infections *in vivo*. Indeed, pharmacological inhibition of ROS production and cationic fluxes, either separately or in combination, results in a similar, drastic, impaired ability of neutrophils to kill *C. albicans* (28). Thus, the efficacy of combinatorial stress-mediated killing of *C. albicans* may explain why this fungus only causes systemic infections in immunocompromised hosts. How does the exquisite sensitivity to combinatorial stress benefit *C. albicans*? It could be speculated that it is nonbeneficial for this pathogen to cause systemic infections as the likely outcome is killing of the host. It seems more likely that *Candida albicans* has evolved as a commensal organism in the human gut and urogenital tracts. In these environments, such combinations of oxidative and cationic stresses may not be encountered. Finally, it is important to note that *C. albicans* is exposed to many other combinations of stresses, in addition to oxidative and cationic stresses, following phagocytosis, such as reactive nitrogen and chloride species, antimicrobial peptides, pH fluctuations, and nutrient limitation (reviewed in reference 43). Therefore, future work will be directed at investigating the impact of other combinations of physiologically relevant stress conditions on killing *C. albicans* and other important fungal pathogens.

## MATERIALS AND METHODS

### Strains and media.

The strains used in this study are listed in Table S1 in the supplemental material. *C. albicans* cells were grown in Tris-buffered yeast extract-peptone-dextrose medium (YPDT [pH 7.4]) (27, 44). Oxidative stress was imposed by treating cells with a range of H_2_O_2_ (Sigma) concentrations as indicated, and cationic stress was imposed with 1 M NaCl. To stress cells with a combination of oxidative and cationic stresses, medium was supplemented with 5 mM H_2_O_2_ plus 1 M NaCl, as detailed previously (27, 28).

### Tagging of Crm1.

To C-terminally tag Crm1, expressed from its native chromosomal locus, with 2 copies of the Myc epitope and 6 His residues, the 3′ region of *CRM1* was amplified by PCR using the oligonucleotide primers Crm1MHPstF (AATGTCTGCAGCAATTATCTGGAGAAGC) and CrmMHPstR (AATGTCTGCAGTTCGTCATCCATTTCAGAAGG) and genomic DNA template. The resulting PCR product was digested with PstI and ligated into the PstI site of CIp-MH-PstI plasmid (31) to generate pCRM1-MH. pCRM1-MH was linearized by digestion with EcoRV to target integration at the *CRM1* locus in SN148 wild-type cells (45) or *hog1*Δ cells (JC45) to generate strains JC1925 and JC1940, respectively. Correct insertion and tagging of Crm1 were verified by PCR and DNA sequencing.

### Cap1 localization.

*C. albicans* cells expressing GFP-tagged Cap1 (JC1060) were prepared as described previously (11, 46). 4′,6-Diamidino-2-phenylindole (DAPI) and GFP fluorescence was captured using a Zeiss Axioscope with a 63× oil immersion objective and the AxioVision imaging system.

### Cap1 phosphorylation and oxidation assays.

To monitor Cap1 phosphorylation, protein extracts were prepared as described previously (47) from exponentially growing *C. albicans* cells expressing 2Myc- and 6His-tagged Cap1 (JC948) before and after treatments with 5 mM H_2_O_2_, 1 M NaCl, or 5 mM H_2_O_2_ plus 1 M NaCl for the indicated times. Twenty-five micrograms of protein was subjected to SDS-PAGE, and Cap1-MH was detected using anti-Myc antibodies (9E10 [Sigma]) (31).

To monitor Cap1 oxidation, protein extracts were prepared under acid lysis conditions and treated with the thiol alkylating agent AMS, as described previously (31). To determine the nature of Cap1 oxidation following combinatorial stress and high ROS levels, protein extracts were obtained under acid lysis conditions and incubated with *N*-ethylmaleimide (NEM [Thermo Scientific, Paisley, United Kingdom]) at a final concentration of 10 mM at 30°C for 30 min in order to block free thiols, precipitated with 1 vol of 20% trichloroacetic acid (TCA) on ice for 30 min, and washed extensively three times with acetone. NEM-labeled samples were resuspended in sample buffer (200 mM Tris-HCl [pH 8], 1% SDS, 1 mM EDTA) supplemented with 20 mM DTT and incubated at 37°C for 60 min in order to reduce the existing disulfides. Acid precipitation and acetone washes were repeated, and protein pellets were resuspended in sample buffer containing methoxy PEG-maleimide (mPEG-Mal) (PEG-maleimide with a molecular weight of 2,000 [Nanocs, Inc., Boston, MA]) at a final concentration of 10 mM at 30°C for 30 min (29) in order to label free thiols that were involved in the formation of disulfides. Acid precipitation and acetone washes were repeated, and samples were treated with 5 U of alkaline phosphatase (New England Biolabs) at 37°C for 1 h to prevent phosphorylation from having an impact on AMS/PEG-maleimide-dependent mobility shifts (26). Samples were subjected to SDS-PAGE on 8% gels under nonreducing conditions, and Cap1-MH was detected as described above.

### Northern blotting.

Northern blotting was performed as described previously (11). Gene-specific probes were amplified by PCR from genomic DNA using oligonucleotide primers specific for *ACT1*, *CAT1*, and *TRR1* (11). RNA levels were quantified using the Typhoon phosphorimaging system (Amersham Biosciences) and Image Quant software (GE Healthcare Life Sciences).

### Detection of intracellular ROS levels.

For intracellular ROS detection, exponentially growing *C. albicans* cells were treated with 20 µM of the fluorescent probe dihydroethidium (DHE [Sigma]) and incubated at 30°C for 45 min in the dark. Cells were washed with phosphate-buffered saline (PBS), sonicated for 15 s, and subjected to fluorescence-activated cell sorting using a FACSAria Fusion (BD Biosciences, Sydney, Australia) with an argon 488-nm laser emitting at 595 nm. Data were analyzed from three independent biological replicates using FlowJo software (TreeStar, Inc., Ashland, OR).

### ChIP-qPCR.

Chromatin immunoprecipitation (ChIP) assays were performed as described by Liu et al. (48), with slight modifications. Briefly, three independent cultures of exponentially growing *C. albicans* JC465 (untagged control strain) and JC948 (Cap1-MH-tagged strain) cells were collected and chromatin fixed by the addition of formaldehyde (1% vol/vol) for 30 min at room temperature with agitation at 20 rpm. Formaldehyde was quenched by the addition of 125 mM glycine at room temperature at 20 rpm for 10 min. Samples were centrifuged, washed twice with ice-cold Tris-buffered saline (TBS) buffer (20 mM Tris-HCl [pH 7.5], 150 mM NaCl), and snap-frozen in liquid nitrogen. The preparation of the total cell extracts and sonication was performed as described previously (48), followed by the overnight immunoprecipitation of the soluble chromatin extracts from both tagged and untagged strains with monoclonal mouse c-Myc antibody (9E10) (Santa Cruz Biotech) coupled to magnetic beads (Dynabeads [Invitrogen Life Technologies]) at 4°C. Beads were washed and reverse cross-linked, and DNA was recovered as described previously (48). The DNA concentrations were quantified using Quant-iT Picogreen double-stranded DNA assay kit (Molecular Probes/Invitrogen) as described previously (48). The DNA concentrations ranged from 0.03 ng/µl to 0.69 ng/µl for the tagged strains and 0.49 ng/µl to 0.72 ng/µl for the untagged strains. Quantitative PCRs (qPCRs) were performed on the Mastercycler ep realplex (Eppendorf) qPCR system using the Takyon ROX Probe MasterMix (Eurogentec) and the conditions described previously (49). Oligonucleotides for the *CAT1*, *TSA1*, and *GLR1* promoter regions (targets) (see Table S2 in the supplemental material) were optimized using Primer3Web software (version 4.0.0.). *ACT1* was used as a control for statistical analyses by *t* test, with *TEF1* (orf19.1435) as a reference for normalization (50). All PCR products had melting curves indicating the presence of a single amplicon. qPCR data were analyzed using the threshold cycle (2^−ΔΔ*CT*^) method (51), and statistical significance was determined using Welsh’s two-sample *t* test.

### Crm1-Cap1 coimmunoprecipitation assay.

Exponentially growing wild-type (JC1925) or *hog1*Δ (JC1940) cells expressing 2Myc- and 6His-tagged Crm1 were harvested before and after stress treatments. Total protein extracts were prepared as described previously (47), and Crm1-MH was immunoprecipitated using anti-Myc (9E10) antibody-coupled agarose (Santa Cruz Biotechnologies), followed by incubation with 200 U of Lambda protein phosphatase (New England Biolabs) for 30 min at 30°C. Precipitated proteins and 5% of protein input were subjected to SDS-PAGE. Coprecipitation of Cap1 was monitored by Western blotting using anti-Cap1 polyclonal antibodies (kindly provided by Scott Moye-Rowley, University of Iowa). Membranes were subsequently probed with anti-Myc antibodies (9E10 [Sigma]) to determine the levels of precipitated Crm1 levels. Quantitative densitometric analysis of Western blots was conducted using ImageJ 1.44 to determine the fold enrichment of Cap1 interaction with Crm1 relative to time zero. Analysis of variance (ANOVA) was used to determine statistically significant differences in Cap1 enrichment levels.

### Detection of RNA Pol II phosphorylation.

To monitor RNA Pol II phosphorylation, protein extracts were prepared as described previously (47) from exponentially growing *C. albicans* cells expressing 2Myc- and 6His-tagged Cap1 (JC948) before and after treatments with 5 mM H_2_O_2_, 25 mM H_2_O_2_, or 5 mM H_2_O_2_ plus 1 M NaCl for the indicated times. Fifteen micrograms of protein was subjected to SDS-PAGE, and Pol II phosphorylation was detected using anti-RNA Pol II phospho S2 (ab5095 [Abcam]) and anti-RNA Pol II phospho S5 (ab5408 [Abcam]) antibodies. Total levels of RNA Pol II were detected using an anti-RNA Pol II CTD repeat antibody (ab817 [Abcam]). Blots were stripped, and Cap1-MH was detected using anti-Myc antibodies (9E10 [Sigma]) (31).

## SUPPLEMENTAL MATERIAL

Figure S1  Quantification of Cap1 levels pre- and post-stress treatment. (A) Cap1 protein levels were analyzed by SDS-PAGE and Western blotting of native extracts prepared from cells expressing Cap1-HM before (ns) and following exposure to 5 mM H_2_O_2_, 1 M NaCl, or combinations of these stresses for 10 min. Blots were stripped and reprobed with an anti-Hog1 antibody as a loading control. (B) Quantification of Cap1 levels. Quantitative densitometric analysis of Western blots from five biological replicates was conducted to determine the relative levels of Cap1 following the stress treatments described above, compared to nonstress (ns) levels. Mean values (±SEM) are shown, and ANOVA was used to determine statistically significant differences in Cap1 levels. No significant differences were observed (*P* > 0.05). Download Figure S1, TIF file, 1.2 MB

Figure S2  Cap1 is differentially oxidized following combinatorial stress. (A) Cap1 mobility was monitored by nonreducing SDS-PAGE and Western blotting of proteins prepared from cells expressing Cap1-HM exposed to 5 mM H_2_O_2_, 1 M NaCl, or combinations of these stresses for 10 min. Disulfide bonds are indicated by the retarded mobility of Cap1 due to AMS binding to DTT-resolved disulfides (compare NEM-DTT- with NEM-DTT-AMS-treated lanes). (B) Cap1 mobility was monitored by nonreducing SDS-PAGE and Western blotting of proteins prepared from unstressed cells (ns) expressing Cap1-HM. Protein extracts were obtained under acid lysis conditions and incubated or not with *N*-ethylmaleimide, which blocks free thiol groups, thus preventing oxidation. Download Figure S2, TIF file, 1.2 MB

Figure S3  The NaCl-enhanced interaction between Cap1 and Crm1 is Hog1 independent and cationic stress specific. (A) Biological replicate of the pulldown shown in Fig. 7B. (B) Hog1 is dispensable for the salt-induced Cap1-Crm1 interaction. Extracts were prepared from *hog1*Δ cells (JC45), and *hog1*Δ cells expressing 2Myc- and 6His tagged Crm1 (JC1940) before and following exposure to 5 mM H_2_O_2_, 1 M NaCl, or combinations of these stresses for 10 min. Coprecipitation of Cap1 was detected as described in the legend to Fig. 7B. The asterisk designates a nonspecific band as seen in *cap1*Δ cells. (C) Sorbitol does not stimulate Cap1 binding to Crm1. Extracts were prepared from wild-type cells expressing 2Myc- and 6His tagged Crm1 (Crm1-MH [JC1925]) before and following exposure to 1 M NaCl for 10 min or 2 M sorbitol for the indicated times. Coprecipitation of Cap1 was detected as described in the legend to Fig. 7B. Download Figure S3, TIF file, 1.9 MB

Figure S4  RNA Pol II phosphorylation is maintained following high-H_2_O_2_ exposure and combinatorial stress. Lysates from cells expressing 2Myc- and 6His-tagged Cap1 (Cap1-MH [JC948]), before (ns) and after the indicated stress treatments were analyzed by Western blotting using antibodies that detect RNA Pol II CTD phosphorylation at serine 2 or serine 5 of the repeat sequence YSPTSPS. Total levels of RNA Pol II were determined using an antibody that recognizes the CTD repeat sequence. As a control, a time zero sample was prepared that was treated with lambda phosphatase prior to loading (+pptase). The positions of nonphosphorylated (RNA Pol II) and phosphorylated (RNA-Pol II S2-P, RNA-Pol II S5-P) Pol II are indicated and are consistent with the predicted molecular mass of *C. albicans* Pol II (192 kDa). The RNA Pol II blot was stripped and reprobed with anti-Cap1 antibodies. This shows that although Cap1 phosphorylation (a marker of active Cap1) is significantly delayed following high-H_2_O_2_ stress and combinatorial stress, RNA Pol II phosphorylation is not reduced following such treatments. This indicates that the defect in Cap1-mediated gene expression is not due to a global inhibition of RNA Pol II activity. Download Figure S4, TIF file, 1.7 MB

Table S1  Strains used in this study. Table S1, DOCX file, 0.02 MB

Table S2  Oligonucleotides used for qPCR. Table S2, DOCX file, 0.01 MB
