# Supplementary figures and images for: Mechanisms Underlying the Delayed Activation of the Cap1 Transcription Factor in Candida albicans following Combinatorial Oxidative and Cationic Stress Important for Phagocytic Potency
Source: mBio. 2016 Mar 29;7(2):e00331-16. doi: 10.1128/mBio.00331-16 (PMC4817257; doi:10.1128/mBio.00331-16)

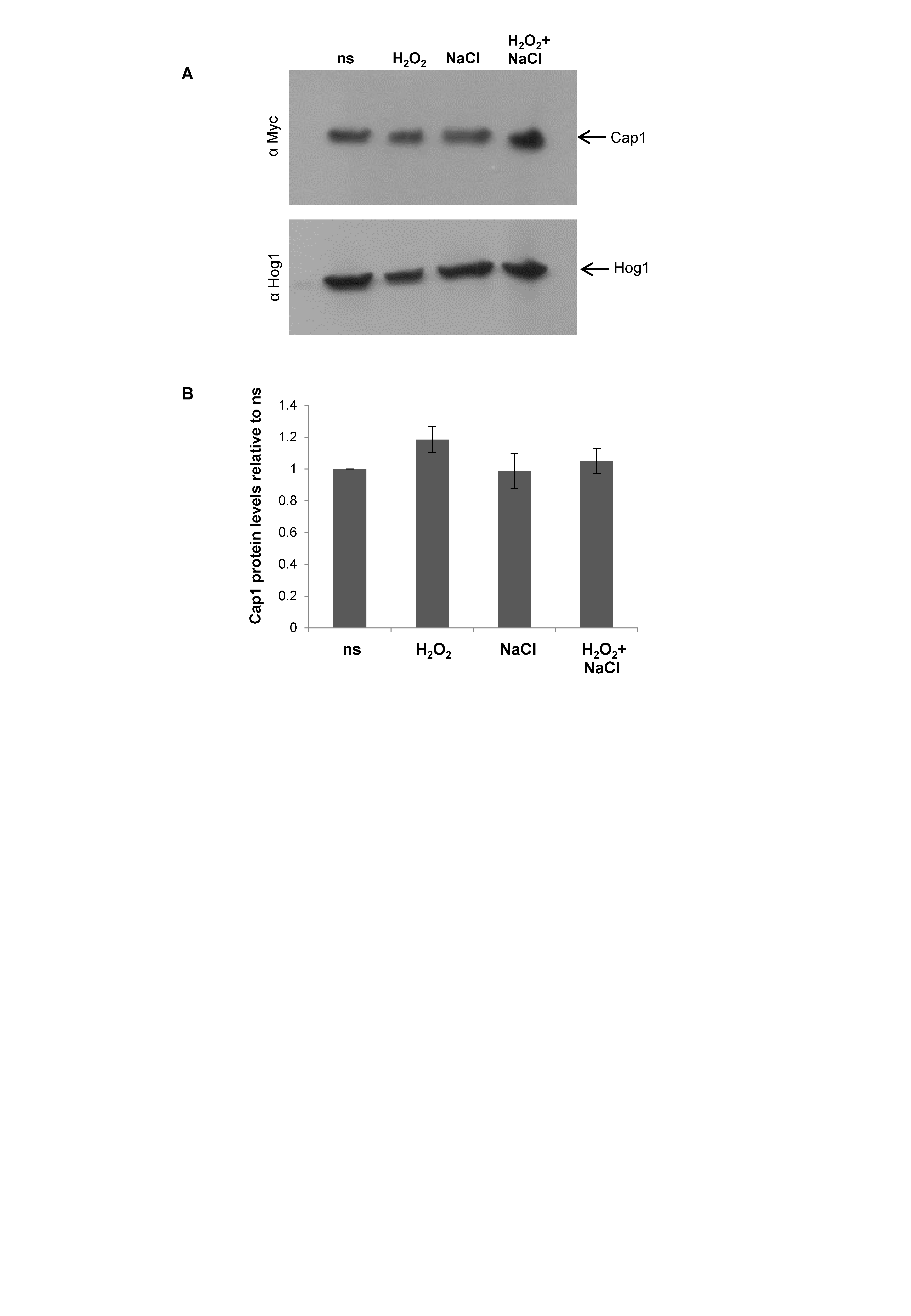

Supplement: Figure S1 — Quantification of Cap1 levels pre- and post-stress treatment. (A) Cap1 protein levels were analyzed by SDS-PAGE and Western blotting of native extracts prepared from cells expressing Cap1-HM before (ns) and following exposure to 5 mM H2O2, 1 M NaCl, or combinations of these stresses for 10 min. Blots were stripped and reprobed with an anti-Hog1 antibody as a loading control. (B) Quantification of Cap1 levels. Quantitative densitometric analysis of Western blots from five biological replicates was conducted to determine the relative levels of Cap1 following the stress treatments described above, compared to nonstress (ns) levels. Mean values (±SEM) are shown, and ANOVA was used to determine statistically significant differences in Cap1 levels. No significant differences were observed (P > 0.05). Download [file mbo002162748sf1.tif]

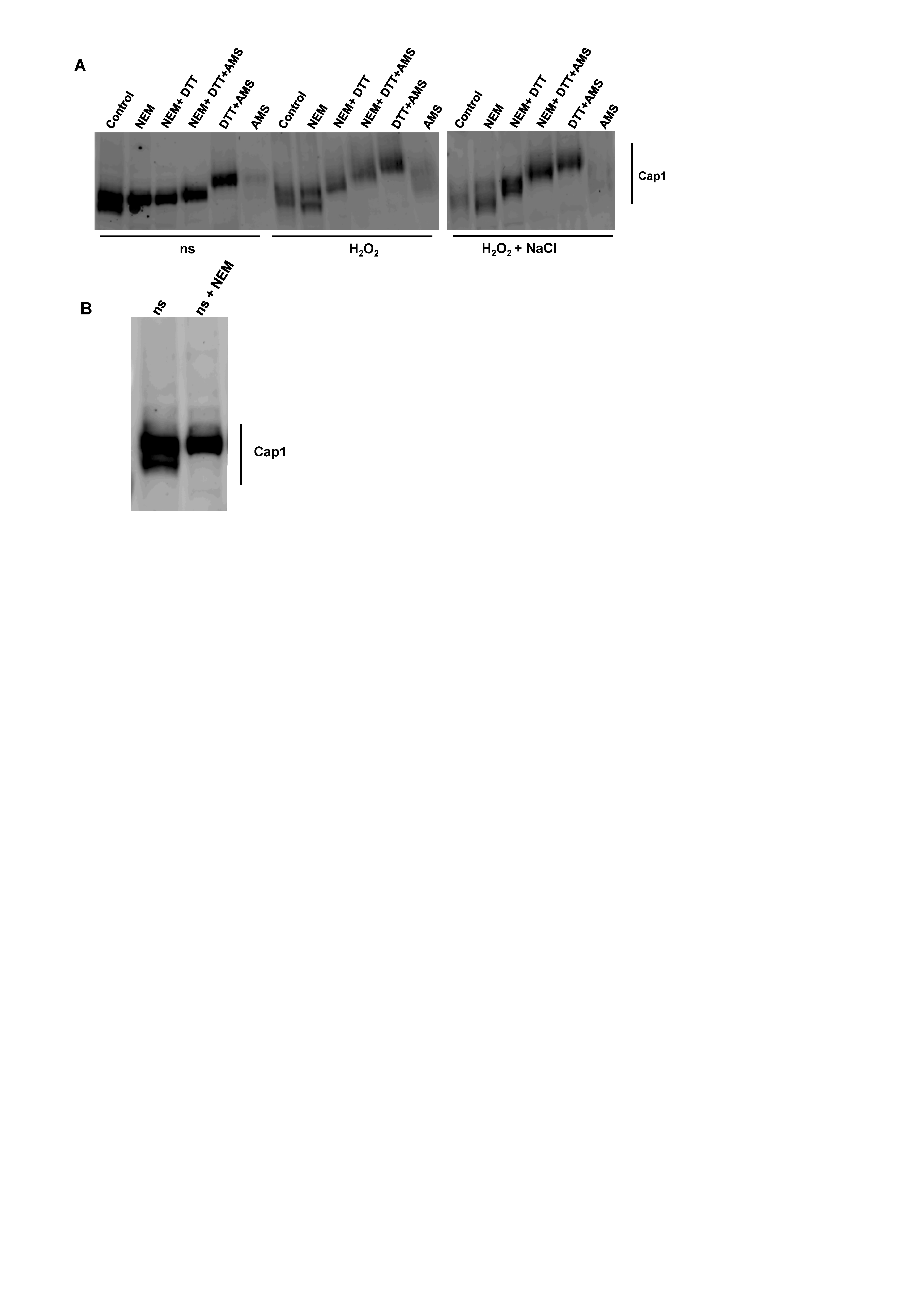

Supplement: Figure S2 — Cap1 is differentially oxidized following combinatorial stress. (A) Cap1 mobility was monitored by nonreducing SDS-PAGE and Western blotting of proteins prepared from cells expressing Cap1-HM exposed to 5 mM H2O2, 1 M NaCl, or combinations of these stresses for 10 min. Disulfide bonds are indicated by the retarded mobility of Cap1 due to AMS binding to DTT-resolved disulfides (compare NEM-DTT- with NEM-DTT-AMS-treated lanes). (B) Cap1 mobility was monitored by nonreducing SDS-PAGE and Western blotting of proteins prepared from unstressed cells (ns) expressing Cap1-HM. Protein extracts were obtained under acid lysis conditions and incubated or not with N-ethylmaleimide, which blocks free thiol groups, thus preventing oxidation. Download [file mbo002162748sf2.tif]

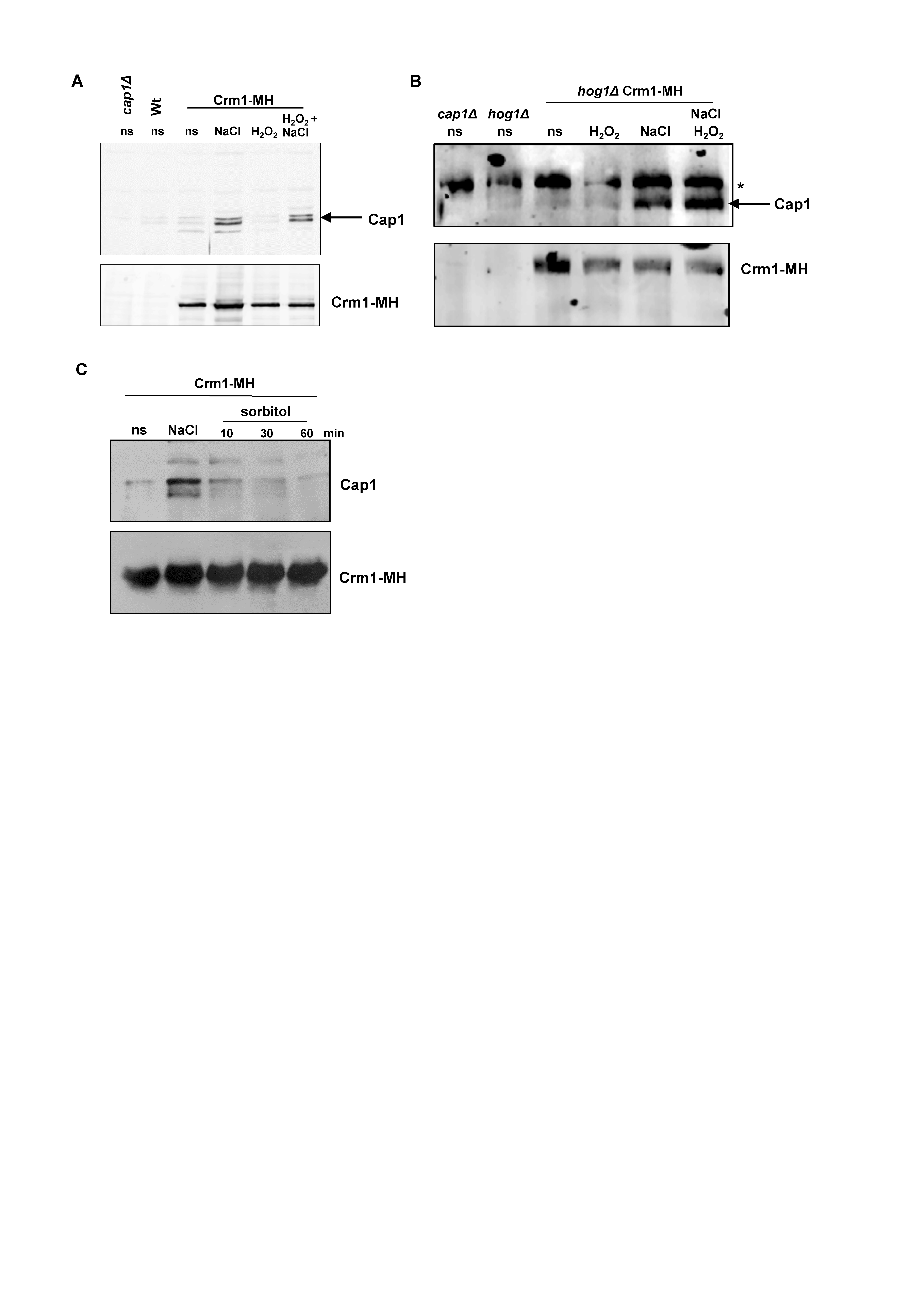

Supplement: Figure S3 — The NaCl-enhanced interaction between Cap1 and Crm1 is Hog1 independent and cationic stress specific. (A) Biological replicate of the pulldown shown in Fig. 7B. (B) Hog1 is dispensable for the salt-induced Cap1-Crm1 interaction. Extracts were prepared from hog1Δ cells (JC45), and hog1Δ cells expressing 2Myc- and 6His tagged Crm1 (JC1940) before and following exposure to 5 mM H2O2, 1 M NaCl, or combinations of these stresses for 10 min. Coprecipitation of Cap1 was detected as described in the legend to Fig. 7B. The asterisk designates a nonspecific band as seen in cap1Δ cells. (C) Sorbitol does not stimulate Cap1 binding to Crm1. Extracts were prepared from wild-type cells expressing 2Myc- and 6His tagged Crm1 (Crm1-MH [JC1925]) before and following exposure to 1 M NaCl for 10 min or 2 M sorbitol for the indicated times. Coprecipitation of Cap1 was detected as described in the legend to Fig. 7B. Download [file mbo002162748sf3.tif]

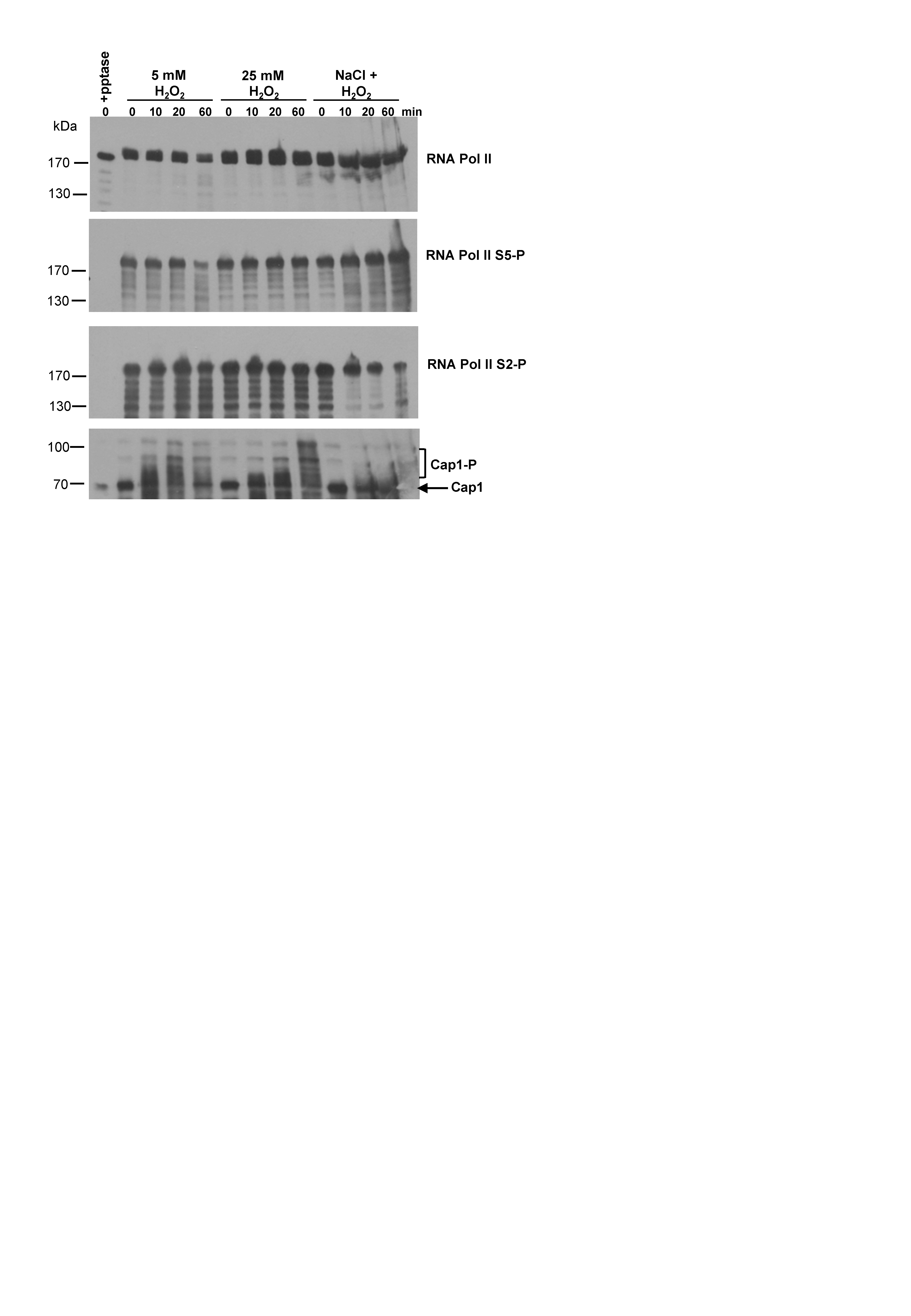

Supplement: Figure S4 — RNA Pol II phosphorylation is maintained following high-H2O2 exposure and combinatorial stress. Lysates from cells expressing 2Myc- and 6His-tagged Cap1 (Cap1-MH [JC948]), before (ns) and after the indicated stress treatments were analyzed by Western blotting using antibodies that detect RNA Pol II CTD phosphorylation at serine 2 or serine 5 of the repeat sequence YSPTSPS. Total levels of RNA Pol II were determined using an antibody that recognizes the CTD repeat sequence. As a control, a time zero sample was prepared that was treated with lambda phosphatase prior to loading (+pptase). The positions of nonphosphorylated (RNA Pol II) and phosphorylated (RNA-Pol II S2-P, RNA-Pol II S5-P) Pol II are indicated and are consistent with the predicted molecular mass of C. albicans Pol II (192 kDa). The RNA Pol II blot was stripped and reprobed with anti-Cap1 antibodies. This shows that although Cap1 phosphorylation (a marker of active Cap1) is significantly delayed following high-H2O2 stress and combinatorial stress, RNA Pol II phosphorylation is not reduced following such treatments. This indicates that the defect in Cap1-mediated gene expression is not due to a global inhibition of RNA Pol II activity. Download [file mbo002162748sf4.tif]
