# Supplementary material for: Mechanisms Underlying the Delayed Activation of the Cap1 Transcription Factor in Candida albicans following Combinatorial Oxidative and Cationic Stress Important for Phagocytic Potency
Source: mBio. 2016 Mar 29;7(2):e00331-16. doi: 10.1128/mBio.00331-16 (PMC4817257; doi:10.1128/mBio.00331-16)
Supplement: Table S1 — Strains used in this study. [file mbo002162748st1.docx]

**Supplementary Table 1.*Candida albicans* strains used in this study.**

| **Strain** | **Genotype** | **Source** |
| --- | --- | --- |
| SN148 | *arg4Δ/arg4Δ,leu2Δ/leu2Δ,his1Δ/his1Δ,ura3Δ::imm434/ura3Δ::imm434 iro1Δ::imm434/iro1Δ::imm434* | (1) |
| JC747 | SN148 + CIp30 (*URA3*, *HIS1*, *ARG4*) | (2) |
| JC948 | SN148 *CAP1-MH-URA3* | (2) |
| JC1060 | SN148 *CAP1-GFP-URA3* | (2) |
| JC1311 | *gpx3Δ::loxP-HIS1-loxP/gpx3Δ::loxP-ARG4-loxP CAP1-MH-URA3* | (3) |
| JC954 | *ypb1Δ::loxP-HIS1-loxP/ybpΔ::loxP-ARG4-loxP CAP1-MH-URA3* | (3) |
| JC1925 | SN148 *CRM1-MH-URA3* | This work |
| JC1940 | SN148 *hog1Δ CRM1-MH-URA3* | This work |

**References**

1. **Noble SM, Johnson AD.** 2005. Strains and strategies for large-scale gene deletion studies of the diploid human fungal pathogen *Candida albicans*. Eukaryot Cell **4:**298-309.

2. **da Silva Dantas A, Patterson MJ, Smith DA, Maccallum DM, Erwig LP, Morgan BA, Quinn J.** 2010. Thioredoxin regulates multiple hydrogen peroxide-induced signaling pathways in Candida albicans. *Mol Cell Biol* **30:**4550-4563.

3. **Patterson MJ, McKenzie CG, Smith DA, da Silva Dantas A, Sherston S, Veal EA, Morgan BA, MacCallum DM, Erwig LP, Quinn J.** 2013. Ybp1 and Gpx3 signaling in *Candida albicans* govern hydrogen peroxide-induced oxidation of the Cap1 transcription factor and macrophage escape. Antioxid Redox Signal **19:**2244-2260.
