# Supplementary material for: Mechanisms Underlying the Delayed Activation of the Cap1 Transcription Factor in Candida albicans following Combinatorial Oxidative and Cationic Stress Important for Phagocytic Potency
Source: mBio. 2016 Mar 29;7(2):e00331-16. doi: 10.1128/mBio.00331-16 (PMC4817257; doi:10.1128/mBio.00331-16)
Supplement: Table S2 — Oligonucleotides used for qPCR. [file mbo002162748st2.docx]

**Supplementary Table 2. Oligonucleotide primers used for qPCR.**

| **Promoter** | **ChIP qPCR Primers (5’-3’)**  **SYBR Green Method** | **Amplicon location relative to translation start site (ATG codon) – Assembly 19** |
| --- | --- | --- |
| *CAT1* | F:CACCACTTATAACCACCCATTTAG  R:GGTCACAGAAATATGGTAGAAGTGA | -990 to -773 (218 bp) |
| *TSA1* | F:GTCTGTTATGCCCAATAGGTAAGAT  R:ATTGGTGAGTGAGAGCTACAATATG | -522 to -317 (206 bp) |
| *GLR1* | F:TATATTGTCCGTCTGTGTGTATGC  R:CGCTCCACAGTATAACTTATCAAAG | -261 to -126 (136 bp) |
| *ACT1* | F:TATGAAAGTTAAGATTATTGCTCCACCAGAAA  R:GGAAAGTAGACAATGAAGCCAAGATAGAAC | +927 to +1,012 (86 bp) |
| *TEF3* | F:GATCACAATTGGGTCCAAGG  R:AGCAGCGGCAATCTTGTTAC | +2,938 to +3,042 (105 bp) |
